# Prevalence of posttraumatic stress disorder (PTSD) in Canada during the COVID-19 pandemic: results from the Survey on COVID-19 and Mental Health

**DOI:** 10.24095/hpcdp.45.1.02

**Published:** 2025-01

**Authors:** Murray Weeks, Danielle Marion, Anne-Marie Robert, R. Nicholas Carleton

**Affiliations:** 1 Centre for Surveillance and Applied Research, Health Promotion and Chronic Disease Prevention Branch, Public Health Agency of Canada, Ottawa, Ontario, Canada; 2 Department of Psychology, University of Regina, Regina, Saskatchewan, Canada

**Keywords:** posttraumatic stress disorder, symptoms, PCL-5, mental health, SCMH, survey

## Abstract

**Introduction:**

This study provides a descriptive overview of the prevalence of posttraumatic stress disorder (PTSD) in Canada, across sociodemographic characteristics, mental health–related variables and negative impacts of the COVID-19 pandemic.

**Methods:**

Data were obtained from cycles 1 and 2 of the Survey on COVID-19 and Mental Health (SCMH), collected in fall 2020 (N = 14689) and spring 2021 (N = 8032). The prevalence of PTSD was measured using the PTSD Checklist for *DSM-5* (PCL-5) Cross-sectional associations were quantified using logistic regression, while controlling for sociodemographic characteristics.

**Results:**

The overall prevalence of PTSD was 6.9%. Factors associated with higher PTSD prevalence were female gender; younger age; lower income (females only); living in an urban area; frontline worker status or not being at work in the past week (males only); fair or poor mental health; a weak sense of community belonging; symptoms of generalized anxiety disorder and major depressive disorder; suicidal ideation; heavy alcohol use; daily cannabis use; increased alcohol and cannabis use since the start of the pandemic; decreased alcohol consumption since the start of the pandemic (males only); concerns about violence in the home; and negative impacts of the pandemic.

**Conclusion:**

PTSD prevalence in Canada varies significantly across sociodemographic groups and is more common among those with indicators of lower mental health and well-being, as well as those more adversely affected by the COVID-19 pandemic. Ongoing and enhanced surveillance of PTSD in Canada is important to better understand and address the burden and impacts of this condition.

**Highlights**

- According to pooled cross-sectional data from fall 2020 and spring 2021, the overall prevalence of PTSD in Canada was 6.9%.
- PTSD prevalence was higher among younger individuals, females, frontline workers, those with lower income (females only) and those living in urban areas.
- PTSD prevalence was higher among individuals with poor or fair mental health or mental health that worsened relative to before the pandemic, a weaker sense of local community belonging, symptoms of anxiety and depression, suicidal thoughts, and heavy or increased use of alcohol and cannabis.
- Individuals who expressed concerns about family violence, particularly females who considered themselves the target of violence, had a higher prevalence of PTSD.
- Prevalence of PTSD increased with the number of reported negative COVID-19 pandemic impacts.

## Introduction

Posttraumatic stress disorder (PTSD) can occur after exposure to a potentially psychologically traumatic event (PPTE), for example, actual or threatened death, natural disasters or sexual assault. Many people in Canada have experienced a PPTE,^1^ but most will not develop PTSD.^2^ PTSD is characterized by post-exposure symptoms that include persistent re-experiencing of the event, frequent avoidance of event reminders, as well as negative thoughts, emotions and behaviours.^3^ A PTSD diagnosis requires post-exposure symptoms that, according to the *Diagnostic and Statistical Manual of Mental Disorders, fifth edition (DSM-5*), cause “clinically significant distress or impairment in social, occupational, or other important areas of functioning.”^3^

PTSD is associated with substantial burdens, including lower quality of life;^4^ higher functional impairment;^5^ and higher prevalence of comorbid mental disorder diagnoses^6^ and alcohol and substance use disorders.^1^ The economic burden of PTSD is unknown, but mental disorders in Canada are associated with billions of dollars per year in lost productivity and health care costs.^7^

PTSD risk factors can be categorized into pre-exposure (e.g. history of mental disorders), peri-exposure (e.g. peri-traumatic dissociation) and post-exposure (e.g. low social support).^8^,^9^ In terms of demographic differences, PTSD is about twice as common among women than men,^1^ is less common in older age groups^10^ and is more common among military personnel^5^ and Veterans,^11^ first responders and other public safety personnel (e.g. correctional workers, firefighters, paramedics, police officers)^12^ and nurses,^13^ among others.

***PTSD prevalence in Canada***

Ongoing surveillance of PTSD prevalence is important for understanding the overall burden and to monitor for trends. Estimates from large nationally representative samples are optimal for addressing these questions. A 2018 systematic review found Canadian PTSD prevalence estimates to be largely dated, based on insufficient data (e.g. nonrepresentative or small samples) and varying greatly in terms of study population (e.g. clinical or general population), PTSD assessment method (clinical assessment, screening tool or self-reported diagnosis) and time frame (e.g. lifetime, past year or past month).^14^ Recent national survey data suggest that the prevalence of a self-reported PTSD diagnosis by a health care professional was 3.4% in 2022 (Public Health Agency of Canada internal analysis using data from the 2022 Mental Health and Access to Care Survey). However, this only includes the treated population who were willing to disclose their diagnosis. 

In 2020, the Survey on COVID-19 and Mental Health (SCMH)^15^ was implemented to examine Canadians’ mental health and well-being in the context of the pandemic. Early findings based on a screening tool (which can identify both diagnosed and undiagnosed individuals) indicated that the prevalence of moderate to severe symptoms of PTSD was 6.3% in 2020 and 7.5% in 2021.^16^

PTSD surveillance is also important for assessing potential impacts of the COVID-19 pandemic. Research conducted in China reported a high prevalence of PTSD during the first month of the pandemic, especially among those who were most impacted,^17^ with one study reporting that more than a quarter of COVID-19 patients screened positive for PTSD within one year of the time that they were affected.^18^ Another study identified risk factors (e.g. economic instability) and protective factors (e.g. timely government action) for PTSD symptoms during the pandemic.^19^ A large population survey in the USA found that 26.3% of adults had symptoms of a trauma- and stressor-related disorder (including PTSD) related to the pandemic.^20^

The current study was designed to use data from cycles 1 and 2 of the SCMH (fall 2020 and spring 2021) to provide a more detailed description of the prevalence of PTSD in Canada, stratifying PTSD prevalence by gender, by several sociodemographic and mental health–related characteristics and by several negative impacts of the COVID-19 pandemic. The current study was also designed to quantify the relationships between these stratification variables and PTSD, while controlling for potential sociodemographic confounders.

## Methods

***Data sources***

Data were collected in fall 2020 (11 September 2020 to 4 December 2020) and in spring 2021 (1 February 2021 to 7 May 2021).^15^ The survey covers non-institutionalized persons aged 18 years and older living in Canada’s 10 provinces and the three territorial capitals, who were not living on reserves or in collective, unmailable, inactive or vacant dwellings. The SCMH used a two-stage sampling design, with the dwelling as the first stage unit and the person within the dwelling as the second stage unit. The SCMH was stratified by province with random sampling of dwellings within each province and within the three territorial capitals. Survey responses were voluntarily completed by electronic questionnaire or through computer-assisted telephone interviews and respondents were informed that their answers would be strictly confidential. The survey response rate was 53.3% (n = 14689) for fall 2020 and 49.3% (n = 8032) for spring 2021. The total sample for both collection periods (n = 18 093) includes those respondents who agreed to share their responses with the Public Health Agency of Canada.

***Measures***

**Sociodemographic variables**

Sociodemographic variables included gender, age group, total household income tertile, highest level of education, area of residence (urban or rural), immigrant status, racialized background and occupation (i.e. frontline work, essential work, other work). 

Immigrants included landed immigrants and nonpermanent residents, and nonimmigrants included people born in Canada. Racialized people were those who did not identify as White or as Indigenous (First Nations, Mtis or Inuit). 

Occupation groups included anyone aged 75 years or younger who worked “at a job or business” in the past week. Frontline workers were defined as people with “the potential to come in direct contact with COVID-19 by assisting those who have been diagnosed with the virus.”^15^ Examples provided were “police officers, firefighters, paramedics, nurses or doctors.”^15^ Essential workers were defined as people working “in a service, facility or in an activity that is necessary to preserving life, health, public safety and basic societal functions of Canadians.”^15^ Examples provided were “employees working in transportation (e.g. public transit, gas stations), financial institutions, health care or as first responders (e.g. police, firefighters, paramedics), pharmacies, childcare, food supply (e.g. grocery stores, truck drivers).”^15^ Frontline workers represented a subset of essential workers; accordingly, the current study excluded frontline workers from the essential worker category to enable comparisons.

**PTSD**

Positive screens for PTSD were assessed using the 20-item PTSD Checklist for *DSM-5*, the PCL-5.^21^ Instead of using the full Life Events Checklist for *DSM-5* (LEC-5),^22^ respondents were asked, “Have you ever experienced a highly stressful or traumatic event during your life?” Respondents were then asked, “Keeping your worst event in mind, over the past month, how often have you been bothered by the following problems?” Respondents rated their symptoms for each PCL-5 item on a 5-point scale (0 = not at all; 1 = a little bit; 2 = moderately; 3 = quite a bit; 4 = extremely). The PCL-5 total score is the sum of the individual item scores. A positive screen for PTSD was based on total scores greater than 32 out of 80.^21^

A more nuanced screen was also applied, wherein the total score threshold was combined with another recommended method where each *DSM-5* criterion is met based on PCL-5 subscale scores.^21^ This more nuanced screening algorithm produced lower overall estimates but a very similar pattern of results (available at https://osf.io/); accordingly, PCL-5 total scores greater than 32 were included. Although aligned with *DSM-5* diagnostic criteria, the PCL-5 does not replace the more comprehensive clinical assessment required for diagnosis. For the sake of simplicity, we use “PTSD prevalence” in this study, despite reference to PCL-5 positive screens.

**Mental health–related variables**

Respondents were asked the following questions about their mental health: (1) “In general, how is your mental health?” with responses dichotomized as “poor/fair” versus “good/very good/excellent”;^23^ and (2) “Compared to before the COVID-19 pandemic, how would you say your mental health is now?” with responses (“much better now,” “somewhat better now,” “about the same,” “somewhat worse now” or “much worse now”) dichotomized as “same or better” versus “worse.”^24^

Respondents were also asked, “How would you describe your sense of belonging to your local community?” Responses were dichotomized as “very strong/somewhat strong” versus “somewhat weak/very weak.”^25^

Symptoms of generalized anxiety disorder (GAD) and major depressive disorder (MDD) were measured using the Generalized Anxiety Disorder Scale (GAD-7)^26^ and the Patient Health Questionnaire (PHQ-9),^27^ respectively, with scores greater than 9 indicating a positive screen on either measure. Respondents were also asked the following two questions: “Have you ever seriously contemplated suicide?” and “Have you seriously contemplated suicide since the COVID-19 pandemic began?” Responses to these facilitated a three-level variable of “lifetime history of suicidal ideation, but not during the COVID-19 pandemic,” “suicidal ideation during the COVID-19 pandemic” and “no history of suicidal ideation.”

Heavy drinking was assessed by asking how often in the past month females had consumed more than 4 drinks in one sitting and males more than 5 drinks in one sitting.^28^ Respondents were also asked how often they consumed cannabis in the past month. Respondents were then asked how their use of alcohol and cannabis had changed over the course of the COVID-19 pandemic when compared to before the pandemic, with potential responses being “increased,” “decreased” and “no change.”

Respondents were asked, “How concerned are you about violence in your home?” Those who reported some level of concern (“somewhat,” “very” or “extremely” vs. “not at all”) were asked who in their household they were concerned was the target of violence. Nonmutually exclusive responses (“self,” “another adult/adults” and “child/children”) were categorized into three groups: “self as target,” “other household member as target” and “no concerns.”)

**COVID-19 impacts**

Respondents were asked, “Have you experienced any of the following impacts due to the COVID-19 pandemic?” Potential answers were “loss of job/income,” “difficulty meeting financial obligations/essential needs,” “death of family/friend/colleague” (i.e. someone close), “feelings of loneliness/isolation,” “emotional distress,” “physical health problems,” “challenges in personal relationships” and “other.” We categorized the total number of reported impacts as “2or fewer,” “3 or 4” and “5 or more.”

***Statistical analyses***

PTSD prevalence estimates were calculated for the overall sample and stratified by gender, for all study variables. Logistic regression analyses estimated associations between the odds of PTSD and these variables. Respondents who entered a gender other than “male” or “female” (i.e. gender-diverse) were not included in gender-stratified analyses because of small counts. Due to the nearly identical methodologies and relative proximity in time, data from the two collection periods were pooled to maximize the sample size for analyses.^29^ However, separate analyses were also conducted for each collection period to enable comparisons.

Logistic regressions were adjusted for the sociodemographic variables listed in the “Measures” section and the collection period (fall 2020 or spring 2021). Proportions and adjusted odds ratios (aORs) were weighted using sampling weights provided by Statistics Canada to ensure the findings were representative of the population in Canada, and adjusted for nonresponse. To account for the effects of the complex survey design of the SCMH, we estimated 95% confidence intervals (CIs) using bootstrap weights for proportions and aORs. Statistically significant aORs were those where unrounded 95% CIs did not include 1. Survey and bootstrap weights were divided in half for pooled analyses.^29^ All analyses used SAS Enterprise Guide version 9.4 (SAS Institute Inc., Cary, NC, US).

## Results

Compared to fall 2020, the spring 2021 sample had a lower proportion of people aged 25 to 34 years, a higher proportion of people aged 35 to 49 years, a higher proportion of essential workers and a lower proportion of other workers (Table 1). A higher proportion of respondents in spring 2021 reported fair/poor mental health, worse mental health since the pandemic began, somewhat or very weak local community belonging, moderate to severe symptoms of MDD, suicidal ideation during the pandemic, and decreased alcohol consumption and cannabis use since the start of the pandemic, whereas a lower proportion reported less than daily heavy drinking. Also, a higher proportion of respondents in spring 2021 reported being affected by the pandemic in terms of the death of someone close, feelings of loneliness/isolation, emotional distress, physical health problems and challenges in personal relationships.

**Table 1:** Sample characteristics for the pooled sample and by data collection period

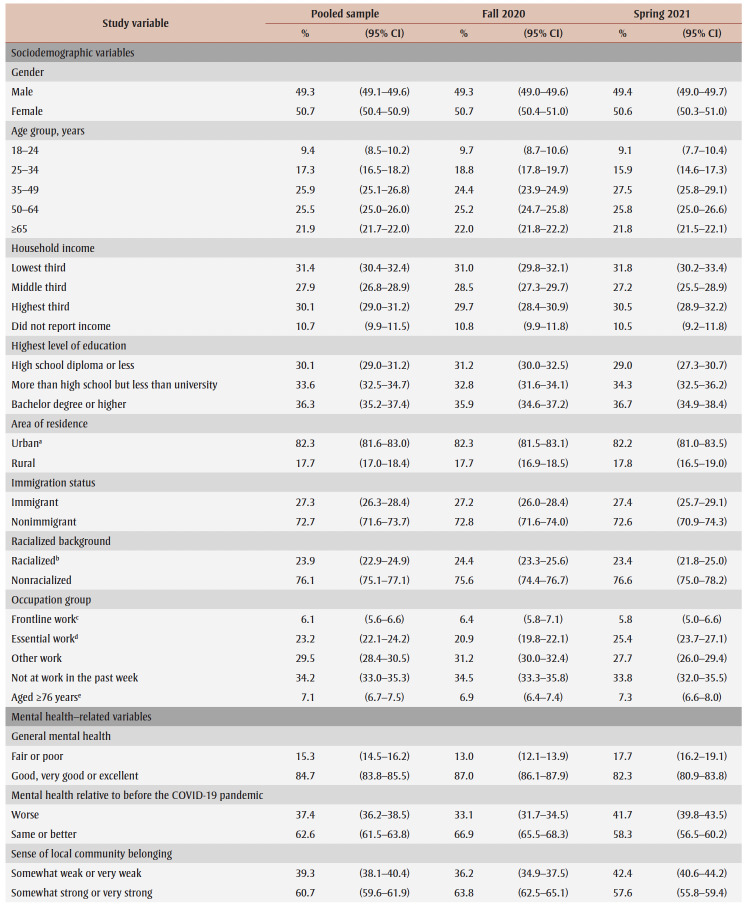 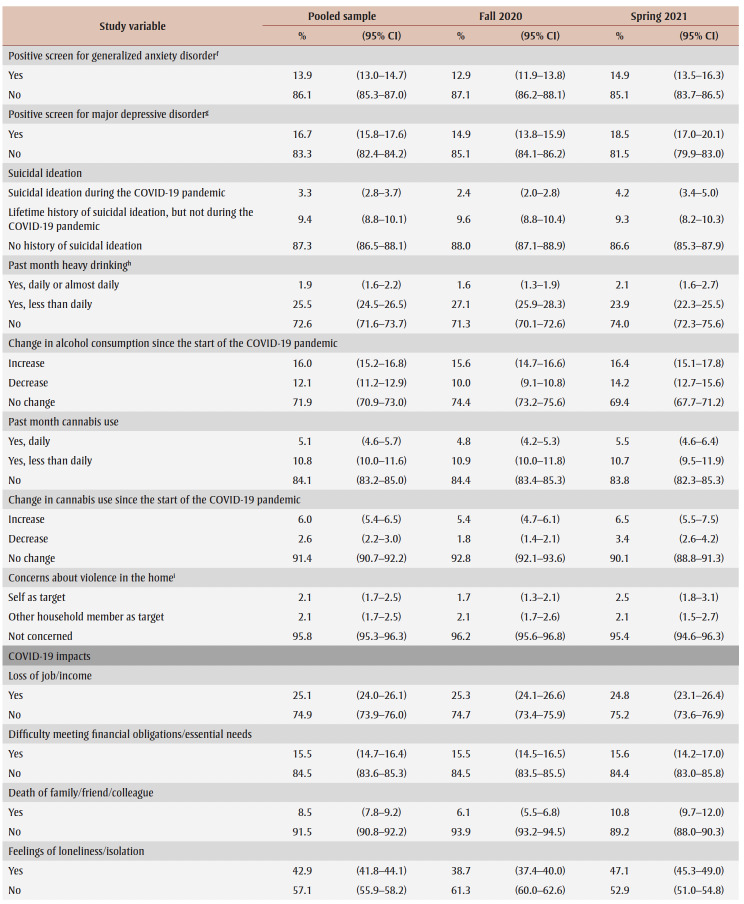 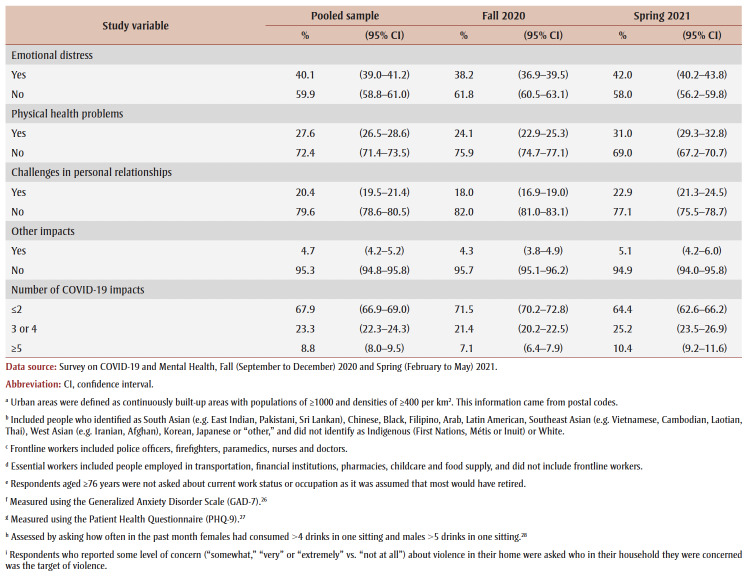

PTSD prevalence in the overall sample was 6.9% (Table 2). Prevalence appeared to be higher (but not statistically significantly) in spring 2021 (7.5%) than in fall 2020 (6.3%). Prevalence was higher among females than males (aOR = 1.8) and among those aged 18 to 64 years than those aged 65 years and older (aOR range: 3.5–6.2). Among females only, prevalence was particularly high among those aged 18 to 24 years (19.9%). 

**Table 2:** PTSD prevalence and adjusted odds ratios, pooled data from fall 2020 and spring 2021, by gender, sociodemographic characteristics, mental health–related variables and impacts of the COVID-19 pandemic

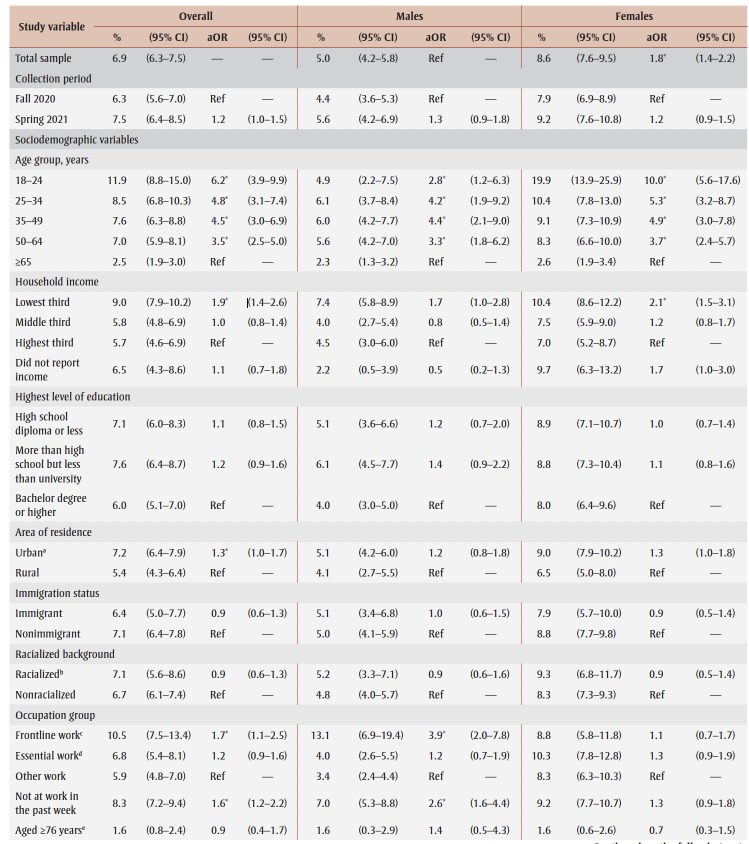 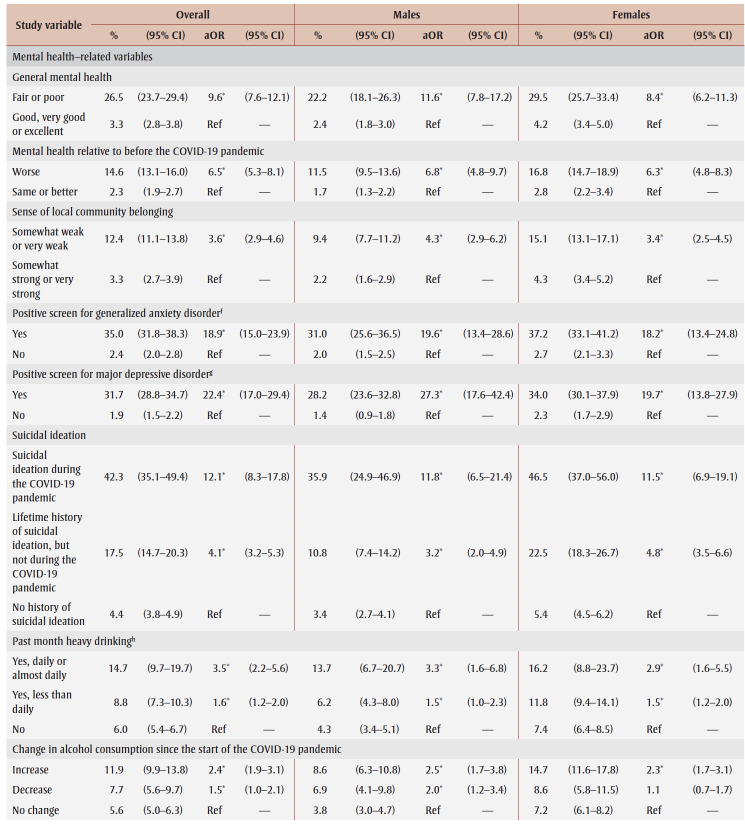 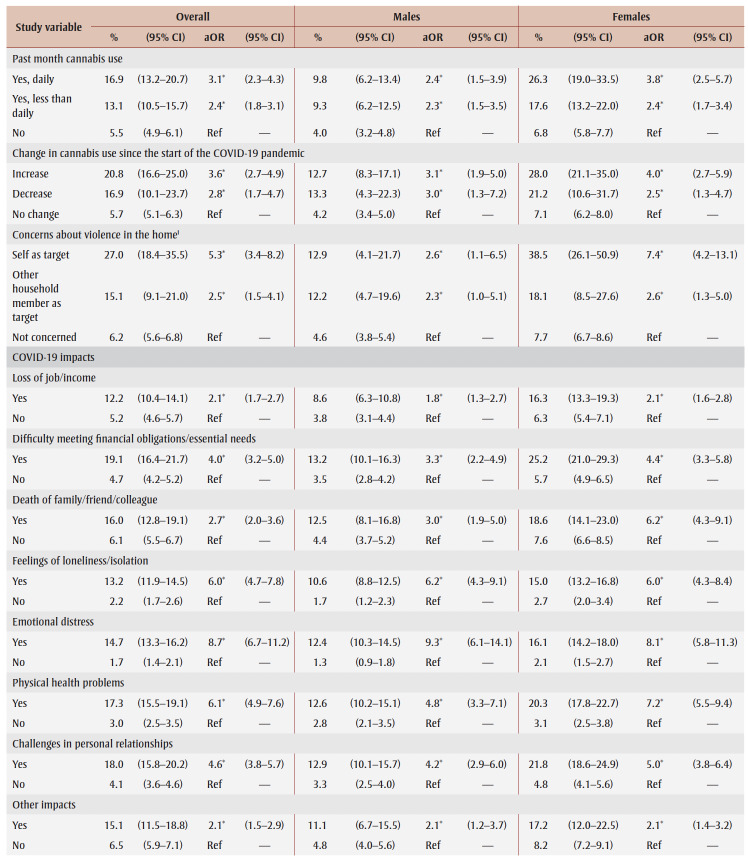 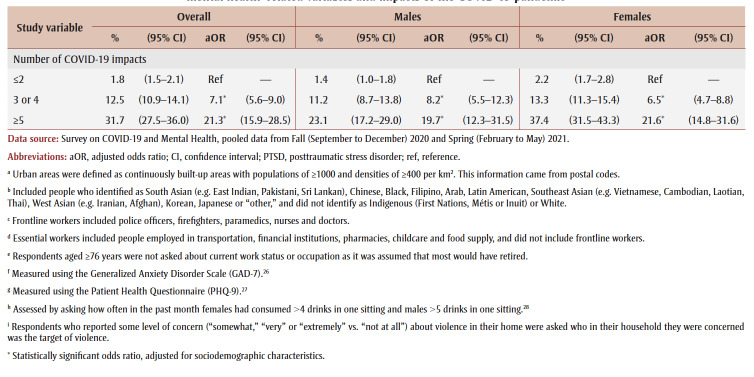

Prevalence was higher among those from the lowest tertile than the highest tertile of household income (aOR = 1.9) and among those living in urban areas (aOR = 1.3). Among males, compared with other workers, prevalence was higher among frontline workers (aOR = 3.9) and those not at work in the past week who were aged 75years or younger (aOR = 2.6).

PTSD prevalence was higher among people who had fair or poor mental health (aOR = 9.6), had worse mental health compared to before the pandemic (aOR = 6.5), had a weaker sense of community belonging (aOR = 3.6), screened positive for GAD (aOR = 18.9) or MDD (aOR = 22.4), had suicidal ideation before (aOR= 4.1) and during (aOR = 12.1) the pandemic; engaged in heavy drinking daily or almost daily (aOR = 3.5) and less than daily (aOR = 1.6), used cannabis daily (aOR = 3.1) and less than daily (aOR = 2.4), increased their alcohol consumption (aOR=2.4) or decreased their alcohol consumption (aOR = 1.5) since the pandemic began, increased their cannabis use (aOR = 3.6) or decreased their cannabis use (aOR = 2.8) since the pandemic began; and reported being concerned about violence in the home where they identified themselves (aOR = 5.3) or another household member (aOR = 2.5) as the target. Identifying oneself as the target of violence was associated with a particularly high PTSD prevalence (aOR= 7.4) among females.

PTSD prevalence was higher among those who had experienced COVID-19 pandemic impacts (aOR range: 2.1 [loss of job/income] to 8.7 [emotional distress]). Prevalence was also higher among those who reported three or four impacts (aOR = 7.1) or five or more impacts (aOR = 21.3) than among those who reported two or fewer impacts.

Tables 3 and 4 show PTSD prevalence for the two collection periods. Among those who experienced job or income loss due to the pandemic, PTSD prevalence was higher in spring 2021 (15.2%) than in fall 2020 (9.3%). In terms of associations between study variables and PTSD, there were no differences in aORs between collection periods. 

**Table 3:** PTSD prevalence and adjusted odds ratios, fall 2020 collection period, by gender, sociodemographic characteristics, mental health–related variables and impacts of the COVID-19 pandemic

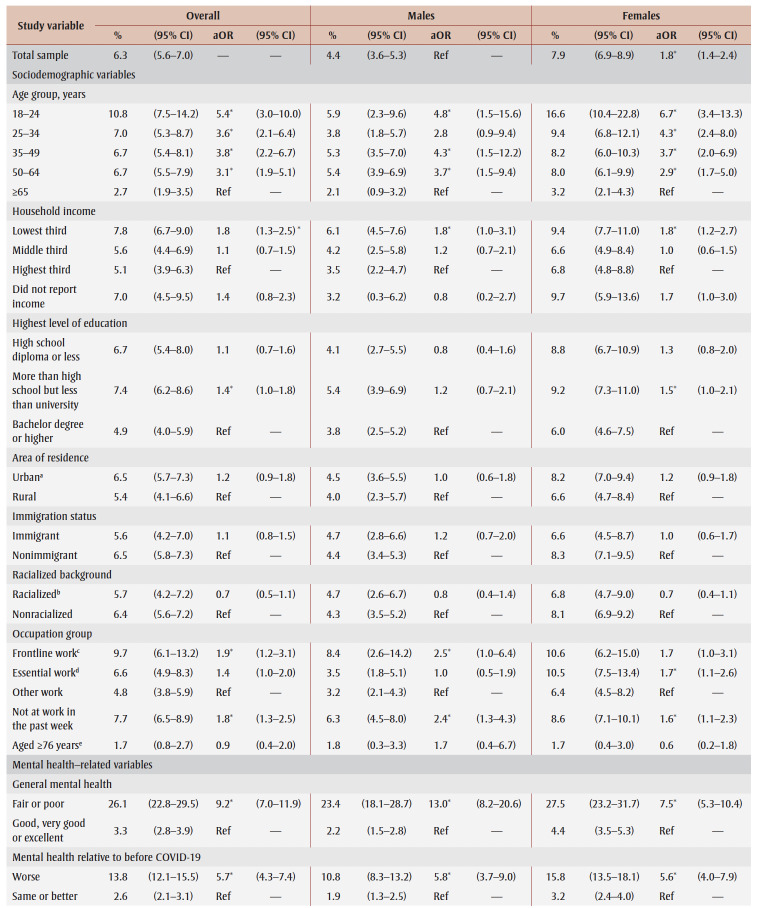 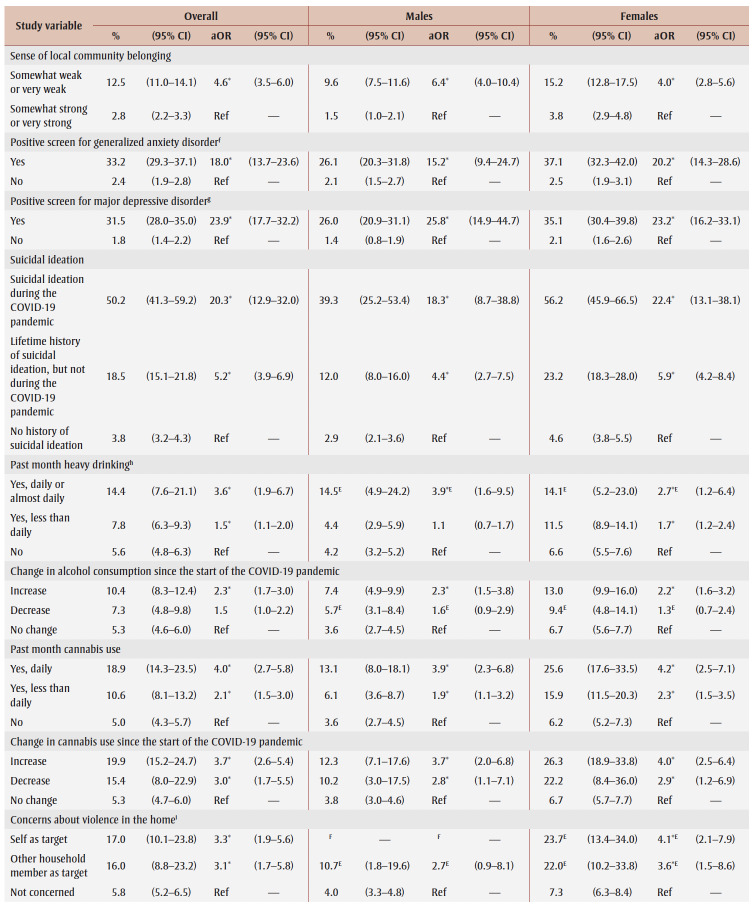 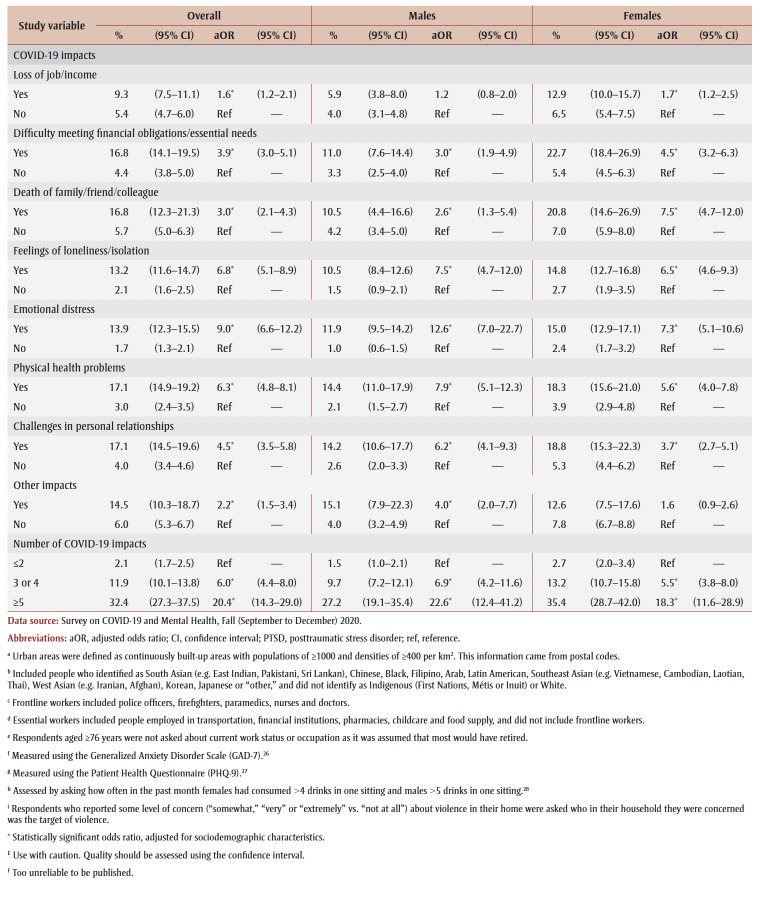

**Table 4:** PTSD prevalence and adjusted odds ratios, spring 2021 collection period, by gender, sociodemographic characteristics, mental health–related variables and impacts of the COVID-19 pandemic

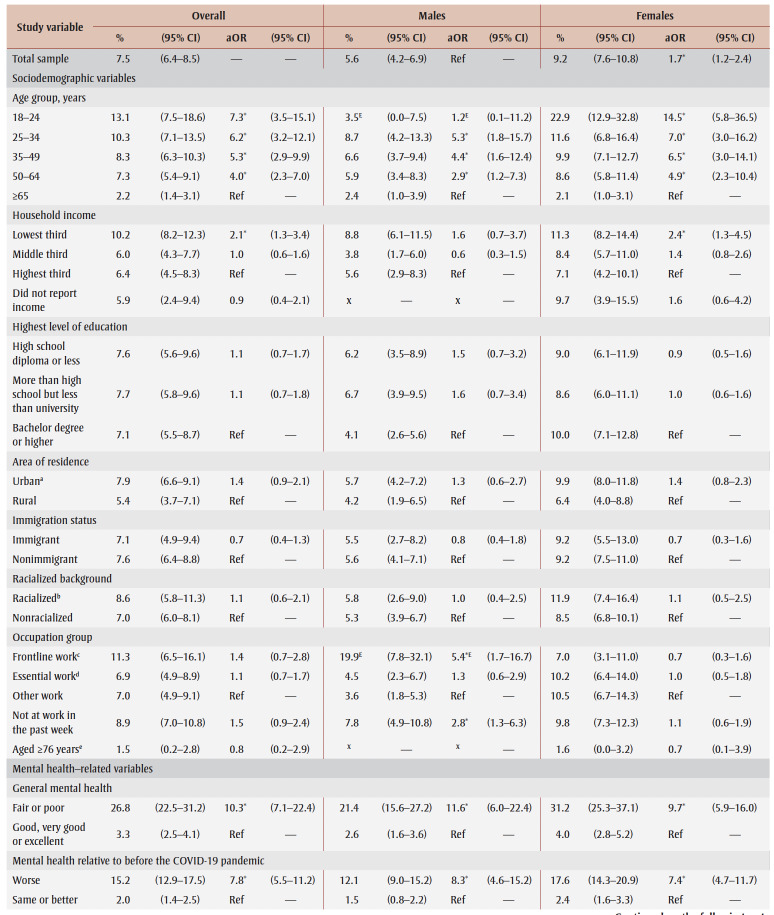 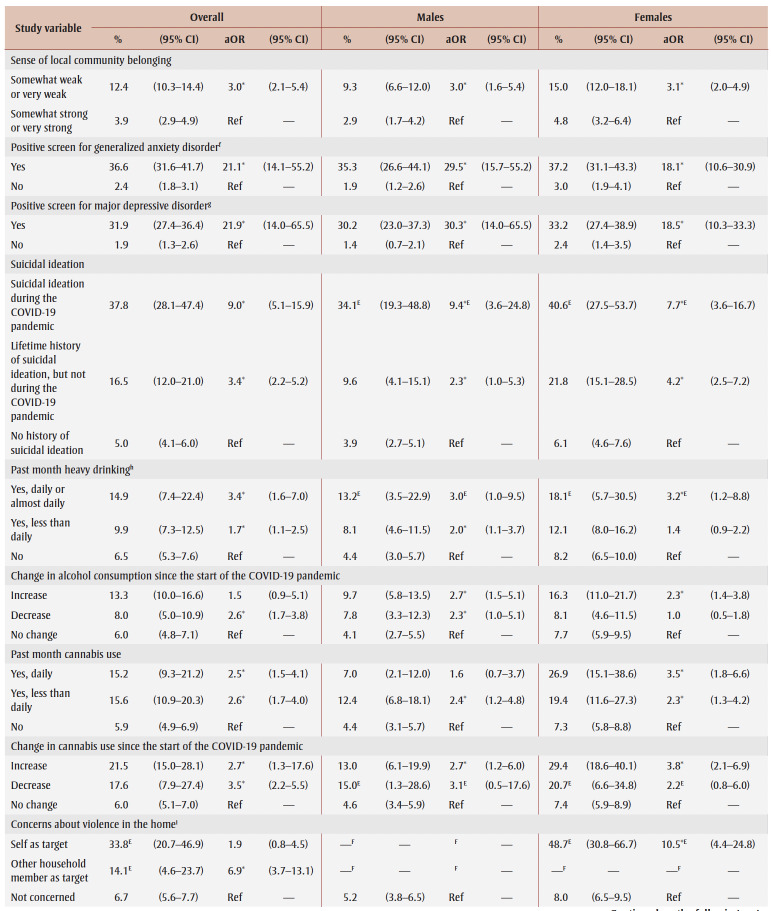 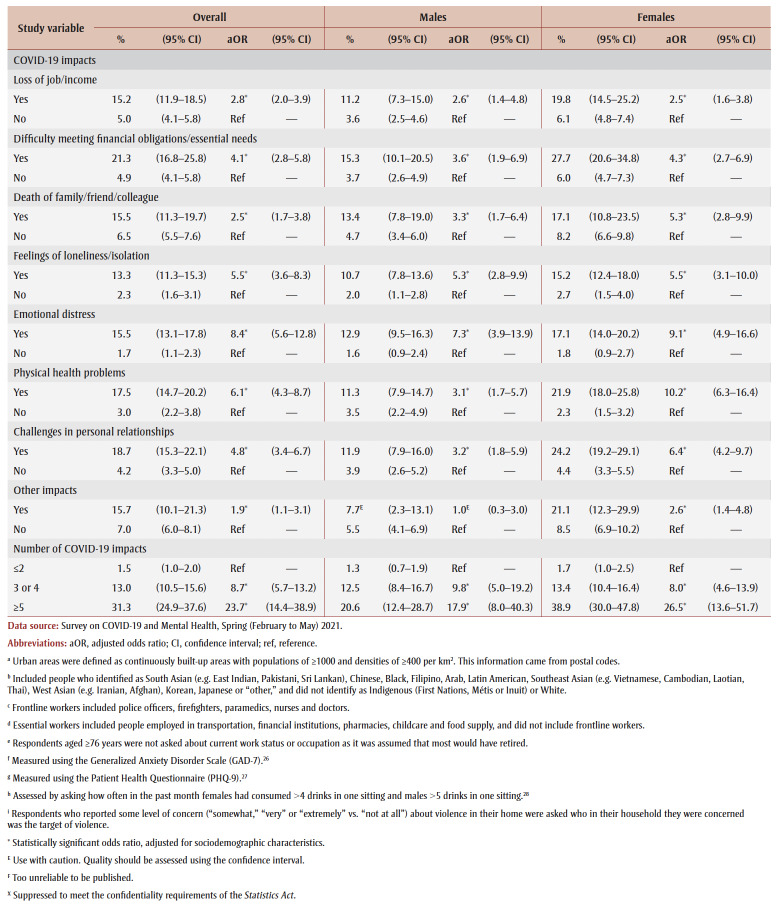

## Discussion

The current study was designed to provide estimates of the prevalence of PTSD in the general population in Canada, across sociodemographic characteristics, mental health–related factors and COVID-19 pandemic impacts in fall 2020 and spring 2021. Contextualizing these results against international data is challenging because of variations in PTSD assessments. A 2017 study in 24 countries (but not including Canada) reported a lifetime PTSD prevalence of 5% in high-income countries, with substantial variation across the countries.^2^ We found the overall prevalence of PTSD to be 6.9%, similar to rates in the USA (6.9%) and Australia (7.3%) in 2017;^2^ however, the Koenen et al. study^2^ took place before the pandemic and used a different assessment method. 

Recent national survey data indicate a notable increase in the prevalence of people self-reporting having received a PTSD diagnosis from a health care professional. In 2021, this prevalence was reported as 5%,^30^ significantly higher than the rates evidenced in 2012 (1.7%) and 2002 (1%).^31^ The results highlight a concerning upward trend in the prevalence of PTSD in Canada and a potential increase in associated burden. This increase in reported PTSD diagnoses could reflect a true increase in PTSD prevalence, increased awareness of PTSD and access to care, greater societal acceptance of and reduced stigma towards PTSD, as well as differences in survey methods. 

The current results align with previous research that show that PTSD is more prevalent among adults who are female,^2^,^3^ younger,^2^,^3^,^32^ living on lower income^2^ and residing in an urban area.^32^ Specifically, the increased risk among younger individuals fits with the broader pattern observed during the pandemic when individuals aged 18 to 34 years disproportionately reported decreased mental health.^33^ Also, sexual assault has a strong association with PTSD,^3^,^34^ but victims of sexual assault are less likely than victims of other crimes to receive support and acknowledgement from others.^35^ Accordingly, the elevated PTSD prevalence among females aged 18 to 24 years is, unfortunately, consistent with expectations.

Frontline workers, including health care workers and public safety personnel, had higher prevalence of PTSD than other occupational groups, which aligns with pre-pandemic research results.^12^ In addition, emerging evidence has highlighted that health care workers experienced higher levels of PTSD during the pandemic.^36^,^37^ Our study results show increased PTSD risk only among male frontline workers, which contradicts previous research findings.^12^,^37^ Further investigation is necessary to gain a deeper understanding of occupational impacts from a gendered perspective.

The results of the current study align with previous research regarding associations between PTSD and neighbourhood social cohesion,^4^ social support^38^,^39^ and symptoms of GAD and MDD.^40^ The results also highlight the substantial level of psychiatric comorbidity among people with PTSD, while further implicating social support as a protective factor.

Evidence of an increased association between PTSD and suicidal ideation relative to before the pandemic is potentially important but may be an artifact of the timing of the PTSD assessment. The current results should be considered in the context of complex interrelationships between PTSD, MDD and suicidal ideation.^41^ Also, PTSD is a risk factor for death by suicide,^42^ but a suicide attempt is also a PPTE that can cause PTSD symptoms.^43^

Higher PTSD prevalence was associated with more frequent heavy drinking, more frequent cannabis use and an increase in alcohol and cannabis use since the pandemic began. The association between PTSD and substance use is complex. Individuals with PTSD often resort to alcohol and cannabis use for symptom management, but misuse of these and other substances can also increase risks for PPTE exposures^44^ and exacerbate PTSD symptoms.^45^ Among females, daily cannabis use was associated with a particularly high PTSD prevalence as compared to less than daily use. The reasons for this gender difference are not clear, but regular cannabis use has been linked to experiences of sexual trauma among females.^46^ While our evidence regarding decreased alcohol consumption and cannabis use may seem counterintuitive, decreased consumption among people who report heavy use, for whom PTSD was more prevalent, may reflect a growing awareness of the problematic nature of their previous consumption.

Our results regarding concerns about violence in the home and PTSD prevalence highlight an important gender difference. PTSD prevalence was similar among males regardless of the target of violence (self versus others). In contrast, among females, PTSD prevalence was noticeably higher when they reported being the target compared to when the target was another household member. This likely reflects the fact that, in Canada, women are significantly more likely than men to be victims of intimate partner violence^47^ and specifically sexual violence,^48^ which in turn is a key predictor of PTSD among abused women.^49^

The degree to which the COVID-19 pandemic has affected PTSD prevalence is unclear. We observed a higher prevalence of PTSD among participants whose mental health had worsened during the pandemic and identified several potential pandemic-related risk factors for PTSD. Across collection periods, we found a general worsening of mental health, a noticeable (though not statistically significant) increase in PTSD prevalence overall and a higher PTSD prevalence associated with job or income loss due to the pandemic. These findings highlight the dynamic nature of the COVID-19 pandemic, including changes over time in terms of its social and economic impacts on society, as well as its potential effects on the prevalence of PTSD. However, the associations are likely bidirectional since pre-existing PTSD symptoms could increase the risk of experiencing these pandemic-related impacts. For instance, the sudden unexpected death of a loved one may account for about one-third of lifetime PTSD cases.^1^

Regardless of causal relationships between pandemic impacts and PTSD, our results show that individuals with PTSD were often substantially affected by the pandemic. Future work will employ more recent Canadian survey data to examine the nature of the PPTEs experienced to provide additional context to prevalence based on screening tools.

***Strengths and limitations***

The main strengths of the current study include the use of a large, nationally representative sample of Canadians, which enhances the generalizability of the findings to the population, as well as the quantification of PTSD risk while controlling for potential confounders.

There are important limitations to consider when interpreting the results. First, the survey was cross-sectional and self-reported, limiting directional statements about effects between variables and facilitating biases such as social desirability or recall bias. Second, certain subpopulations were excluded from the SCMH, limiting generalizability to the entire Canadian population. Third, there are other important intersections of the population that could be explored in terms of PTSD. Our study only identified intersections of study variables with gender and thus could not identify more specific demographic subgroups that could be identified as at-risk. Fourth, the use of a single question to establish the experience of a PPTE, rather than using the LEC-5,^22^ may have affected the PTSD prevalence estimates. Finally, although we used a recommended cut-point for scoring the PCL-5, this method may incorrectly identify PTSD cases (i.e. false positives) due to a lack of focus on clinical criteria. Indeed, sensitivity analyses showed that our alternative scoring method resulted in a significantly lower overall prevalence estimate (5.6%). We have provided these results are available as supplementary materials at https://osf.io/gvbn6/?view_only=c031ae386f364de4a1064 d7bb6d9c8ea to provide context and promote discussion regarding best practices for the use of the PCL-5 to measure PTSD prevalence.

## Conclusion

The current study provides an overview of PTSD prevalence in Canada across many different characteristics, identifies groups at increased risk and highlights the interplay of the negative impacts of the COVID-19 pandemic and PTSD symptoms. The current results may have important implications for policy makers, because they shed light on the burden of PTSD in Canada and they can help guide the development of targeted interventions and support systems for high-risk groups, including in the context of a public health event.

## Acknowledgements

The Public Health Agency of Canada and Statistics Canada designed the Survey on COVID-19 and Mental Health (SCMH), and Statistics Canada implemented it. The data in this paper are based on the SCMH cycles 1 and 2, from fall 2020 and spring 2021.

## Funding

The Public Health Agency of Canada provided all funding for this study.

## Conflicts of interest

The authors have no conflicts of interest to disclose.

## Authors’ contributions and statement

MW: Conceptualization, formal analysis, data curation, writing – original draft, writing – review & editing.

DM: Investigation, writing – original draft, writing – review and editing.

AMR: Writing – review and editing.

RNC: Writing – review and editing.

The content and views expressed in this article are those of the authors and do not necessarily reflect those of the Government of Canada.
